# Dynamical Casimir–Polder force on a two-level atom with superposition state in a cavity comprising a dielectric

**DOI:** 10.1038/s41598-020-68546-6

**Published:** 2020-07-20

**Authors:** Yumei Long, Wentao Wang, Xue Zhang, Hui Yang, Taiyu Zheng

**Affiliations:** 1 Center for Quantum Sciences and School of Physics, Northeast Normal University, Changchun, 130024 China; 2 Center for Advanced Optoelectronic Functional Materials Research, Key Laboratory for UV-Emitting Materials and Technology of Ministry of Education, Northeast Normal University, Changchun, 130024 China; 3 School of Science, Changchun University of Science and Technology, Changchun, 130022 China

**Keywords:** Theoretical physics, Quantum optics

## Abstract

We study the dynamical Casimir–Polder force on a two-level atom with different initial states in the one-dimensional dielectric cavity with output coupling, and obtain the analytical expression of the expectation value of dynamical Casimir–Polder force. Results show that the expectation values of dynamical Casimir–Polder force may be affected by the initial states of the atom. Moreover, the expectation value of Casimir–Polder force may vanish at some special atomic positions by properly selecting the initial state of the system. The effects of different relative dielectric constants and the cavity size on the expectation value of Casimir–Polder force are also discussed.

## Introduction

The existence of electromagnetic vacuum fluctuations has caused many quantum effects, such as Casimir–Polder force^1,2^. The Casimir–Polder force is the long-range interaction between neutral polarizable particles and macroscopic objects, which is successfully observed in experiments^3^ and has received considerable attentions^4–7^ due to its potential application in basic physics and designing of quantum devices^6,8–11^. For instance, the Casimir–Polder force has covered various configurations such as trapping cold atoms near surfaces^12–16^, quantum reflection^17–20^, graphene^21^, Bose-Einstein condensates^17,22^, and carbon nanotubes^23–25^. At finite temperatures, the key correlations between these interactions and the topological and magnetoelectric properties of interacting objects are highlighted^26,27^.

In recent years, many studies have focused on the Casimir–Polder force on atoms when they start from ground or excited states^28–31^. In Ref.^31^ and^4^, the authors investigate the dynamcial Casimir–Polder force on a two level atom placed before a ideal metal plate starting from the ground state and the excited state, respectively. Results show that the static Casimir–Polder force is always attractive for the ground state condition, while the static Casimir–Polder force is either attractive or repulsive for the excited condition. At some special atom-wall distances, the static Casimir–Polder force can vanish for the excited condition. Besides, the static Casimir–Polder force for the excited-atom condition is much greater than that of the ground-atom condition.

In this paper, we will investigate the effect of initial states on the dynamical Casimir–Polder force acting on a two-level atom in a caivity with output coupling^32^. The analytical expression of the expectation value of dynamical Casimir–Polder force on atom starting from the superposition state will be obtained. And the effects of the dielectric and cavity size will be discussed.

The paper is organized as follows: in “Model and Hamiltonian” section, we describe the model and the Hamiltonian of the system. In “Calculation of the expectation value of dynamical Casimir–Polder force” section we will calculate the expectation value of dynamical Casimir–Polder force. In “Dynamical Casimir–Polder force on an initially general superposition state atom” section we will discuss the dynamical Casimir–Polder force on an initially general superposition-state atom, and in “Conclusions” section we will summarize our results.

## Model and Hamiltonian

**Figure 1:**
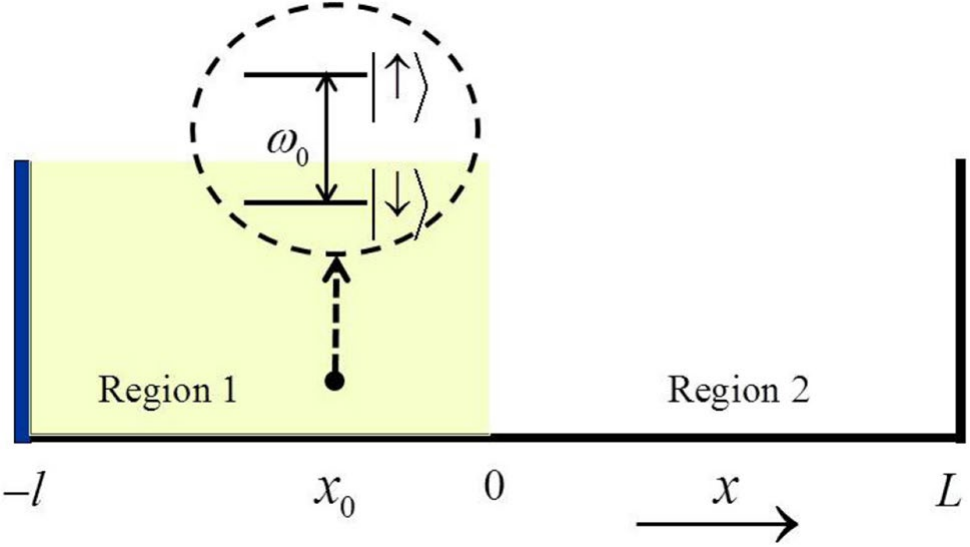
(Color online) The schematic diagram of the structure of a two-level atom located in the one-dimensional dielectric cavity (Region 1). The cavity is embedded in a larger ideal cavity with ideal conductor plates at both ends. The auxiliary cavity (Region 2) acts as an environment.

We consider a one-dimensional cavity which is filled with a dielectric (Region 1 shown in Fig. 1). The wall at $x=-l$ is an ideal-conductor plate. The other wall at $x=0$ is no coating and allows the environment (Region 2 shown in Fig. 1) couples to the cavity. A two-level atom is located at $x_{0}$ in the cavity. The Hamiltonian for the system within the dipole approximation can be written as^32–34^

1 $$\begin{aligned} H= & {} H_{0}+H_{int}, \end{aligned}$$

2 $$\begin{aligned} H_0= & {} \hbar \Omega S_{z}+\sum \limits _j{\hbar \omega _{j}b_{j}^{\dag }b_{j}}, \end{aligned}$$

3 $$\begin{aligned} H_{int}= & {} -e\vec {r}\cdot \vec {E}=-i\sum \limits _j{g_{j}(S_++S_-)(b_j-b_j^\dag )}, \end{aligned}$$

We can defined the parameter $g_{j}$ in Eq. (3)

4 $$\begin{aligned} g_{j}=\hbar \mu \sin [k_{1j}(x_0+l)]\times \left\{ \frac{\omega _{j}}{\hbar \varepsilon _{1} L}{{\frac{2k_{1j}}{k_{2j}}\sum \limits _{n=0}^\infty {\frac{1}{1+\delta _{0,n}} \left( -\frac{k_{1j}-k_{2j}}{k_{1j}+k_{2j}}\right) ^n}\cos (2nk_{1j}l)}}\right\} ^{\frac{1}{2}}, \end{aligned}$$

where $\mu$ is the dipole operator component in the polarization of the field. “1” and “0” are the dielectric constants of the dielectric and vacuum, respectively. $S_{z}, S_{+}$ and $S_{-}$ are the operators of the atomic system, where $S_z=1/2({\left| \uparrow \right\rangle \left\langle \uparrow \right| -\left| \downarrow \right\rangle \left\langle \downarrow \right| })$, $S_+ =\left| \uparrow \right\rangle \left\langle \downarrow \right|$ and $S_- =\left| \downarrow \right\rangle \left\langle \uparrow \right|$. $\left| \uparrow \right\rangle$ and $\left\langle \downarrow \right|$ are the excited and ground states of the atom. $b_j^\dag$ and $b_{j}$ are the creation and annihilation operators for the jth mode of the field with the frequency $\omega _{1j} = c_1 k_{1j}$ and $[b_j ,b_{j'}^\dag ]=\delta _{jj'}$. where $c_1$ is the speed of the light in the dielctric. The superscript $i=1$ or 2 represents to the region 1 or region 2, respectively. $\delta _{0,n}$ is the Kronecker delta.

## Calculation of the expectation value of dynamical Casimir–Polder force

In the above section, we have analyzed the electromagnetic field in the cavity and the Hamiltonian for the system. In the following, we will calculate the expectation value of dynamical Casimir–Polder force ${{\overline{F}}}(x,t)$ via the expectation value of second-order interaction-energy shift $\Delta \overline{E}^{(2)}$ of the system. The Casimir–Polder force is the negative derivative of the second-order energy shift with respect to $x_0$:

5 $$\begin{aligned} {\overline{F}}(x,t)=-\frac{\partial \Delta {\overline{E}}^{(2)}}{\partial x_0}. \end{aligned}$$

In order to get the expectation value of second-order energy shift $\Delta {\overline{E}}^{(2)}$, we will first obtain the Heisenberg equations of the field and atomic operators, and then solve the equations at the zeroth and first orders. According to perturbation theory^31,35^, the equations of $b_{j}\left( t\right)$ and $S_+ \left( t\right)$ are obtained

6 $$\begin{aligned} b_{j}(t)= & {} e^{-i\omega _{j}t}b_{j}(0)+e^{-i\omega _{j}t}g_{nj}[S_{+}(0)\zeta (\omega _{j}+\Omega ,t)+S_{-}(0)\zeta (\omega _{j}-\Omega ,t)], \end{aligned}$$

7 $$\begin{aligned} S_{+}(t)= & {} e^{i\Omega t}S_{+}(0)+2e^{i\Omega t}S_{z}(0)\sum \limits _j{g_{j}[b_{j}^\dag (0)\zeta (\omega _{j}-\Omega ,t)-b_{j}(0)\zeta ^{*}(\omega _{j}+\Omega ,t)]}, \end{aligned}$$

where $\zeta \left( {x,t}\right)$ is defined as

8 $$\begin{aligned} \zeta \left( {x,t}\right) =\int _0^t{e^{ixt}}dt=\frac{e^{ixt}-1}{ix}. \end{aligned}$$

The expectation value of second-order energy shift $\Delta {\overline{E}}^{(2)}$ can be derived by using the perturbation theory^31^.

9 $$\begin{aligned} \Delta {\overline{E}}^{(2)}=\frac{\left\langle \varphi \right| H_I^{(2)}(t)\left| \varphi \right\rangle }{2}, \end{aligned}$$

Here $\left| \varphi \right\rangle$ is the superposition state of the system, and

10 $$\begin{aligned} \left| \varphi \right\rangle =\cos (\theta )\left| \downarrow ,0\right\rangle +\sin (\theta )\left| \uparrow ,0\right\rangle , \theta \in [0,\frac{\pi }{2}]. \end{aligned}$$

That is, the atom may be in the excited state or ground state at *t*=0, and the probability of being in the ground state is $cos^2\theta$, while the probability of being in the excited state is $sin^2\theta$. We evaluate the expectation value of second-order energy shift $\Delta {\overline{E}}^{(2)}$ by substituting Eqs. (6) and (7) into Eq. (3). $H_{int}^{(2)}(t)$ is expressed as

11 $$\begin{aligned} H_{int}^{(2)}(t)= & {} -\frac{i}{\hbar }\sum \limits _{nj} {g_{j}^2}[S_+(0)e^{i\Omega t}+h.c]\times \{e^{-i\omega _j t}[\zeta (\omega _j+\Omega ,t)S_+(0)+\zeta (\omega _j-\Omega ,t)S_-(0)]-h.c\} \nonumber \\&-\frac{2i}{\hbar }S_z(0)\sum \limits _{njn'j'}{g_{j}g_{j'}} \{e^{- i\Omega t}[\zeta ^*(\omega _{j'}-\Omega ,t)b_{j'}(0)-\zeta (\omega _{j'}+\Omega ,t)b_{j'}^\dag (0)]+h.c\} \times[b_j(0)e^{-i\omega _j t}-h.c]. \end{aligned}$$

Then the expectation value of second-order energy shift $\Delta {\overline{E}}^{(2)}$ is obtained

12 $$\begin{aligned} \Delta {\overline{E}}^{(2)}=\frac{\left\langle \varphi \right| H_{int}^{(2)}(t)\left| \varphi \right\rangle }{2}=-\frac{1}{\hbar }\sum \limits _{nj}g_{j}^2\{{\cos ^2\theta \cdot \frac{[1 -\cos [(\omega _j+\Omega )t]]}{\omega _j+\Omega }-\sin ^2\theta \cdot \frac{[1-\cos [(\omega _j-\Omega )t]]}{\omega _j-\Omega }}\}. \end{aligned}$$

We can calculate $\Delta {\overline{E}}^{(2)}$ by using the method of Ref.^31,32^, and obtain the expectation value of Casimir–Polder force by Eq. (5). The expression of the expectation value of Casimir–Polder force can be obtained as follows: For $t>2(x_0+l)/c_{1}$,

13 $$\begin{aligned} {\overline{F}}(t,\theta )= & {} \frac{\mu ^{2}}{4\pi \varepsilon _{1}}\{\frac{\tau ^{2}}{2}[\pi \cos (z_{0})-2\cos (2\theta )h_{2>}(z_{0},t)]-\cos (2\theta )F_{0}(t) \nonumber \\&+\sum \limits _{n=1}^{\infty }\left( -\frac{\sqrt{\varepsilon _{r}}-1}{\sqrt{\varepsilon _{r}}+1}\right) ^{n}\times \{\frac{\tau ^{2}}{2}[\pi \cos (z_{1})-2\cos (2\theta )h_{2>}(z_{1},t)] \nonumber \\&-\frac{\tau ^{2}}{2}[\pi \cos (z_{2})-2\cos (2\theta )h_{2>}(z_{2},t)]-\cos (2\theta )F_{1}(t)\}\}. \end{aligned}$$

where we define the notations as follows:

14 $$\begin{aligned} F_0 (t)= & {} \tau ^{2}h_{1}(z_{0})- \frac{{\sin (\Omega t)}}{2}h_3 \left( \frac{{z_0 }}{\tau },t\right) - \frac{\tau }{2}\cos (\Omega t)h_4 \left( \frac{{z_0 }}{\tau },t\right) , \end{aligned}$$

15 $$\begin{aligned} F_1 (t)= & {} \tau ^{2}[h_{1}(z_{1})-h_{1}(z_{2})]-\frac{\sin (\Omega t)}{2}\left[ h_3 (\frac{z_1}{\tau },t) + h_3 \left( - \frac{z_2}{\tau },t\right) \right] - \frac{\tau }{2}\cos (\Omega t)\left[ h_4 \left( \frac{z_1}{\tau },t\right) \right. \nonumber \\&\left. + h_4 \left( - \frac{z_2}{\tau },t\right) \right] , \end{aligned}$$

16 $$\begin{aligned} h_{1}(z)= & {} \frac{1}{z}-\mathrm {C}i(z)\sin (z) + \cos (z)\mathrm {S}i(z), \nonumber \\ h_{2<} (z,t)= & {} \sin (z)[\mathrm {C}i(z + \Omega t) + \mathrm {C}i( z - \Omega t)]- \cos (z)[\mathrm {S}i(z + \Omega t) + \mathrm {S}i( z- \Omega t)-\pi ] ,\nonumber \\ h_{2>} (z,t)= & {} \sin (z)[\mathrm {C}i(z + \Omega t) + \mathrm {C}i( -z + \Omega t)]- \cos (z)[\mathrm {S}i(z + \Omega t) - \mathrm {S}i( -z + \Omega t)] ,\nonumber \\ h_3 (z,t)= & {} \frac{1}{(z + \frac{v_{1}t}{2})^2 } - \frac{1}{(z-\frac{v_{1}t}{2})^2 }, \nonumber \\ h_4 (z,t)= & {} \frac{1}{z + \frac{v_{1}t}{2}} + \frac{1}{z-\frac{v_{1}t}{2}}. \end{aligned}$$

Herein, we define $\tau =2\Omega \sqrt{\varepsilon _r}/c, c=3\times 10^8\,\hbox {m}/\hbox {s}, z_0=\tau (x_0+l),z_1=\tau (x_0+l+nl)$ and $z_2=-\tau (x_0+l-nl)$. $\mathrm {S}i(z)$ and $\mathrm {C}i(z)$ represent the sine integral function and the cosine integral function, respectively.

## Dynamical Casimir–Polder force on an initially general superposition state atom

**Figure 2:**
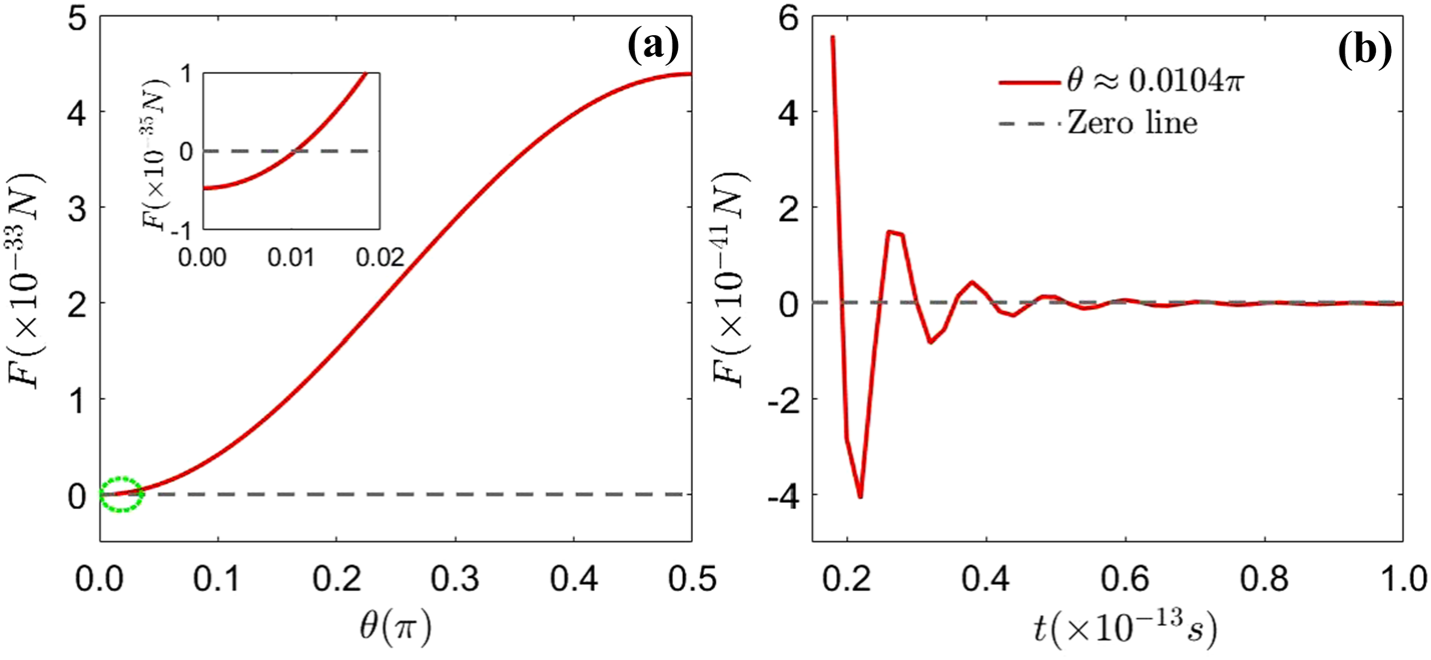
(Color online) Box (**a**) indicates the evolution of the expectation value of Casimir–Polder force with $\theta$ for $t=2\times 10^{-13}$s. When $\theta =0.0104\pi$, the expectation value of Casimir–Polder force equals zero. Box (**b**) shows the time evolution of the expectation value of Casimir–Polder force for the initial state $\theta \approx 0.0104\pi$. The parameters are: $\mu =6.31\times 10^{-30}\,\hbox {cm}, l=2\times 10^{-7}\,\hbox {m}$, $x_0=-0.5\times 10^{-7}\,\hbox {m}, \varepsilon _{r}=1.44$ and $\Omega =1\times 10^{16}\,\hbox {Hz}$.

In this section, we focus on finding the characters of the expectation value of Casimir–Polder force on an initially general superposition state atom. Figure 2a shows that the expectation value of Casimir–Polder force is a function of $\theta$. As is seen in Fig. 2,the expectation value of Casimir–Polder force increases with $\theta$, which shows that the expectation values of dynamical Casimir–Polder force may be affected by the initial state of the atom. In Fig. 2b, for $\theta \approx 0.0104\pi$, we can see that the expectation value of Casimir–Polder force shows oscillations and it reaches a steady zero value for a long time. That is, at special atomic positions, the expectation value of Casimir–Polder force may change from negative value to positive value with different values of $\theta$.

**Figure 3:**
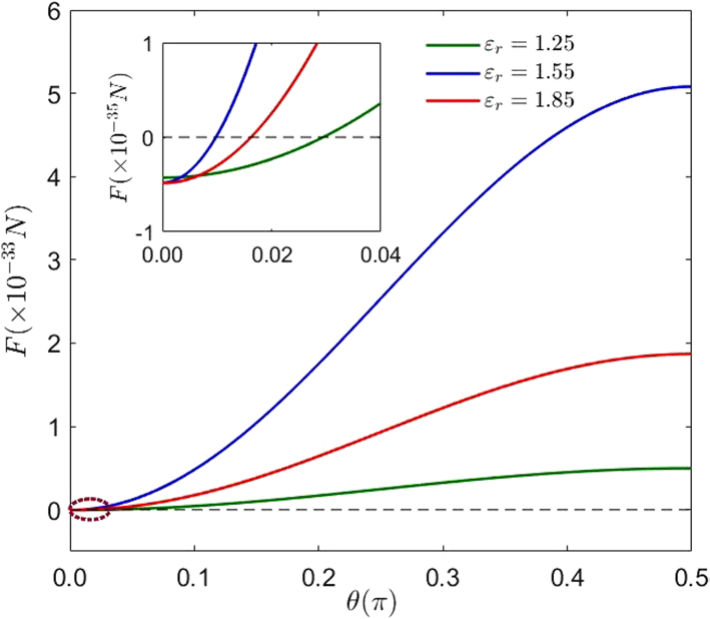
(Color online) The evolution of the expectation of the Casimir–Polder force corresponding to the different relative dielectric constants with $\theta$. The parameters are: $\mu =6.31\times 10^{-30}\,\hbox {cm}, l=2\times 10^{-7}\,\hbox {m}$, $x_0=-0.5\times 10^{-7}\,\hbox {m}$ and $\Omega =1\times 10^{16}\,\hbox {Hz}$.

**Figure 4:**
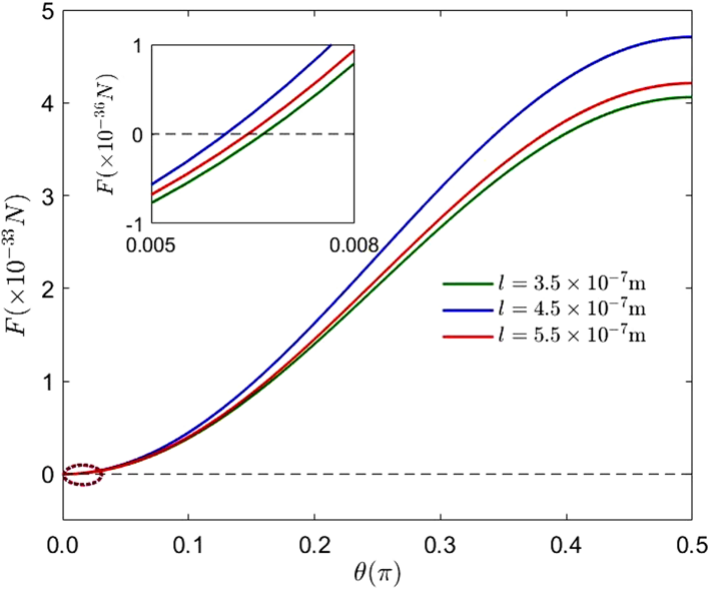
(Color online) The evolution of the expectation of the Casimir–Polder force corresponding to the different cavity lengths with $\theta$. The parameters are: $\mu =6.31\times 10^{-30}\,\hbox {cm}, x_0+l=1.5\times 10^{-7}\,\hbox {m}$, $\varepsilon _{r}=1.44$ and $\Omega =1\times 10^{16}\,\hbox {Hz}$.

To further investigate the effect of the relative dielectric constant and the cavity length on the expectation value of Casimir–Polder force, we describe the plots of the Casimir–Polder force with different values of the relative dielectric constant and the size of cavity versus $\theta$, as shown in Figs. 3 and 4, respectively. In Fig. 3 reveals the effect of different relative dielectric constants on the Casimir–Polder force. The green curve, the blue curve and the red curve indicate the case where the relative dielectric constant is 1.25, 1.55 and 1.85, respectively. The intersection of the red curve ($\varepsilon _{r}=1.85$) and the green curve ($\varepsilon _{r}=1.25$) with the zero line is on the right side of the blue curve ($\varepsilon _{r}=1.55$), which indicates that the initial state which can make the expectation value of the Casimir–Polder force zero may be affected by the relative permittivity of the dielectric.

Figure 4 investigates the effect of different cavity lengths on the expectation of the Casimir–Polder force. The green curve, the blue curve and the red curve indicate the case that the cavity length is $l=3.5\times 10^{-7}\,\hbox {m}, l=4.5\times 10^{-7}\,\hbox {m}$ and $l=5.5\times 10^{-7}\,\hbox {m}$, respectively. The intersection of the red curve ($l=5.5\times 10^{-7}\,\hbox {m}$) and the green curve ($l=3.5\times 10^{-7}\,\hbox {m}$) with the zero line is on the right side of the blue curve ($l=4.5\times 10^{-7}\,\hbox {m}$), which indicates that the initial state which can make the expectation value of the Casimir–Polder force zero may be affected by the size of cavity.

## Conclusions

In this paper, we have calculated the expectation value of dynamical Casimir–Polder force on a two-level atom starting from the different initial states in the one-dimensional cavity with output coupling by using the perturbation theory, and have obtained the analytical expression of the expectation value of dynamical Casimir–Polder force.

We have observed the relationship between the expectation value of Casimir–Polder force and the initial state of the atom, that is, the expectation values of dynamical Casimir–Polder force may be affected by the initial state of the atom. By selecting the proper initial state, the expectation value of Casimir–Polder force may vanish at some special atomic positions. The relative permittivity and the size of the cavity may also affect the expectation value of the Casimir–Polder force.
